# Automated, Noncontact Intraocular Pressure Home Monitoring after Implantation of a Novel Telemetric Intraocular Pressure Sensor in Patients with Glaucoma: A Feasibility Study

**DOI:** 10.1155/2018/4024198

**Published:** 2018-12-06

**Authors:** Antonis Koutsonas, Peter Walter, David Kuerten, Niklas Plange

**Affiliations:** Dept. of Ophthalmology, RWTH Aachen University, Aachen, Germany

## Abstract

**Purpose:**

Reliable and regular assessment of intraocular pressure (IOP) is important for the monitoring of patients with glaucoma. The purpose of this study was to evaluate the feasibility of a novel system for the automated, noncontact measurement of IOP.

**Patients and Methods:**

A first-generation telemetric IOP sensor was previously implanted in the ciliary sulcus of six patients with open-angle glaucoma during cataract surgery. Using this technology, automated noninvasive tonometry may be performed in a home setting. In the present study, a modified sleep mask and a modified eyepatch with incorporated coil antennae for measurements during nighttime and daytime, respectively, were tested on a single patient.

**Results:**

In this feasibility study, the 24 h wear of the prototype measuring apparatus was well tolerated. Three sequences of 24 h IOP measurements with at least 200 IOP measurements per day were performed (Sequence 1: mean 19.6 ± 2.7 mmHg, range 13.4–28.7 mmHg; Sequence 2: mean 21.0 ± 3.0 mmHg, range 13.1–30.5 mmHg; Sequence 3: mean 19.9 ± 2.4 mmHg, range 12.6–27 mmHg).

**Conclusions:**

For the first time, repeated and automated 24-hour measurements are possible using a prototype noncontact reading system after implantation of a novel telemetric IOP sensor in patients with glaucoma.

## 1. Introduction

Glaucoma is a chronic disease requiring lifelong monitoring and treatment. For successful disease management, a high degree of self-discipline and motivation on the patient's part is necessary. In addition, the treating ophthalmologist must provide an individualized monitoring and patient care concept. Adherence to glaucoma therapy is challenged by numerous factors [1, 2]. In many cases, patients remain asymptomatic for a long time, until the development of a severe loss of the visual field and visual acuity associated with advanced stages of the disease.

Approximately 60 million adults aged >40 years suffer from glaucoma worldwide, with more than eight million of those being bilaterally or legally blind [3].

Glaucoma is a complex disease with a multifactorial pathogenesis. Increased intraocular pressure (IOP) is considered to be the greatest risk factor for glaucomatous optic neuropathy [4]. The exact role of IOP in the course of the disease is not entirely understood. Clinical studies have pointed out that short- and long-term fluctuations in IOP may be an independent risk factor for the progression of glaucoma [5, 6]. However, this assumption remains controversial [7].

Single measurements of IOP are regularly performed in day-to-day clinical practice. Ophthalmologists attempt to cover the possible spectrum of IOP fluctuations by performing measurements at different times during the day. Nevertheless, this method provides limited information and does not capture IOP fluctuations occurring between measurements or during nighttime. Patients with unclear progression of glaucomatous damage under seemingly well controlled IOP may benefit from the availability of additional data on IOP.

The combination of noncontact tonometry with self-tonometry assessment in a method that could provide safe, reliable, and repeatable measurements of IOP may assist ophthalmologists in better understanding the role of IOP fluctuation in the progression of glaucoma. Evidence from studies on other chronic diseases (e.g., arterial hypertension) has shown a beneficial effect of self-monitoring on disease control and patient adherence [8, 9]. Therefore, self-tonometry may exert a positive effect on patient adherence and disease management.

The original one-year results [10], along with the recently published long-term follow-up safety report, showed good safety and tolerability following the implantation of a telemetric IOP sensor in patients with glaucoma [11]. However, concerns regarding the accuracy of the sensor and reliability of IOP measurements have been expressed. The main results of the original one-year study and its long-term follow-up are summarized below:

- In the initial postoperative days, four out of the six patients included in the study developed significant, sterile inflammation in the anterior chamber. There were no indications of a prolonged immunological reaction to the sensor, as the inflammation resolved completely within nine days. Assuming the occurrence of an intraocular reaction to the mechanical stress of the implantation procedure, the inflammation was most likely caused by the size of the sensor.
- In the one-year study results, there was a poor correlation between the data from the IOP sensor and those of GAT with strong fluctuations. This poor correlation persisted during the long-term follow-up.
- A mild-to-moderate pupillary distortion was observed in all patients after surgery. This pupillary distortion remained stable over time (except in Patient 4), without further deterioration reported during the long-term follow-up.
- Single IOP sensor measurements were successfully performed at all times and for all patients during the one-year study, as well as during the long-term follow-up.
- In the long term, there were no incidents of late-onset endophthalmitis, chronic inflammation, pain, corneal edema, pupillary block, angle closure, retinal detachment, bleeding, macular edema, and dislocation of the intraocular lens or the telemetric pressure sensor. However, a mild rotation of the sensor was observed in two patients.
- There was no evidence of sensor-related progression of glaucoma during the long-term follow-up.
- During the long-term follow-up, shift phenomena were observed in the telemetric sensor data of four patients. In some cases, the telemetric measurements displayed on the reading unit showed negative values or values approaching zero.

In the present study, we assessed the feasibility of a novel system for automated, noncontact, frequent measurement of IOP during daytime and nighttime. This new approach may provide large amount of data on IOP without the requirement of manual measurements.

## 2. Materials and Methods

A ring-shaped telemetric IOP sensor (ARGOS generation 1, Implandata Ophthalmic Products GmbH, Hannover, Germany) was tested in a prospective, single-center, one-year, pilot clinical trial (ARGOS generation 1 study; Deutsches Register Klinischer Studien [German Clinical Trials Register] DRKS00003335; www.germanctr.de). The telemetric IOP sensor was inserted into the ciliary sulcus (without any suture or other specific fixation) of six patients with glaucoma during planned cataract surgery and after implantation of the intracapsular lens.

The study protocol was reviewed and approved by the institutional ethics committee and conducted in accordance with the principles of the Declaration of Helsinki.

The specifications of the intraocular telemetric sensor have been described previously [10, 12]. A brief summary of the properties of the sensor is provided below.

The ring-shaped telemetric IOP sensor is a miniature device with eight pressure-sensitive capacitors included in a single application-specific integrated circuit combined with a circular microcoil antenna. Changes in the IOP result in mechanical deflections of a capacitor membrane, leading to a change in the capacity. This reflects the absolute IOP, irrespective of the position of the sensor within the eye. The sensor is powered by a high-frequency field emitted from the same reading device transmitting the sensor data. The reading device is held in front of the eye during IOP measurements (measurement duration less than 2 s) and displays an error message if the distance between the sensor and the eye is more than 5 cm. If this distance is less than 5 cm, the reading device displays a valid value. This device has not yet been approved by the United States Food and Drug Administration; however, it was approved by the CE in May 2017.

Based on the positive safety profile of the sensor, offering painless and simple measurements of the IOP, the investigators decided to conduct an automated, noncontact, home monitoring pilot study over several days, initially including a single patient.

Measurements were performed approximately three years after the initial implantation of the intraocular sensor. A 73-year-old male subject included in this study was suffering from normal tension glaucoma, with an unaltered IOP-lowering treatment (two agents) since the implantation procedure.

**Figure 1:**
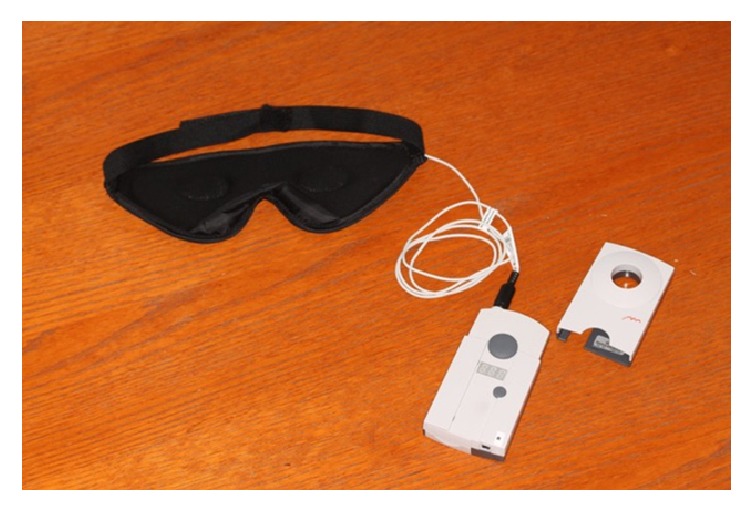
A modified reading device that can automatically perform IOP measurements with 5 min intervals was connected to a prototype sleep mask.

**Figure 2:**
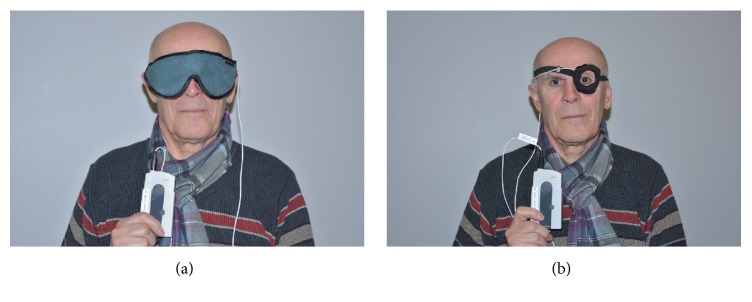
(a) The coil antenna of the reading device was incorporated in the prototype sleep mask, positioned at a short distance from the eye for nighttime measurements. (b) For daytime measurements, the coil antenna was incorporated in a modified eyepatch.

In this study, a modified reading device that can automatically perform IOP measurements with 5 min intervals was connected to a prototype sleep mask (Figure 1). The coil antenna of the reading device was incorporated in the prototype sleep mask, positioned at a short distance from the eye for nighttime measurements (Figure 2(a)). For daytime measurements, the coil antenna was incorporated in a modified eyepatch (Figure 2(b)). The participating patient provided written informed consent for the publication of these images.

## 3. Results

Using this approach, more than 850 measurements were performed over a period of five consecutive days. During three consecutive days, three sequences of 24 h IOP measurements (with ≥200 IOP measurements per day) with only few interruptions were performed. On day 6, the patient returned to the clinic to submit the apparatus and share his experience with the investigator.

In this feasibility study, the 24 h wear of the prototype measuring apparatus was well tolerated. IOP measurements throughout daytime and nighttime were performed with no serious impairment of daytime activities and quality of sleep.

**Figure 3:**
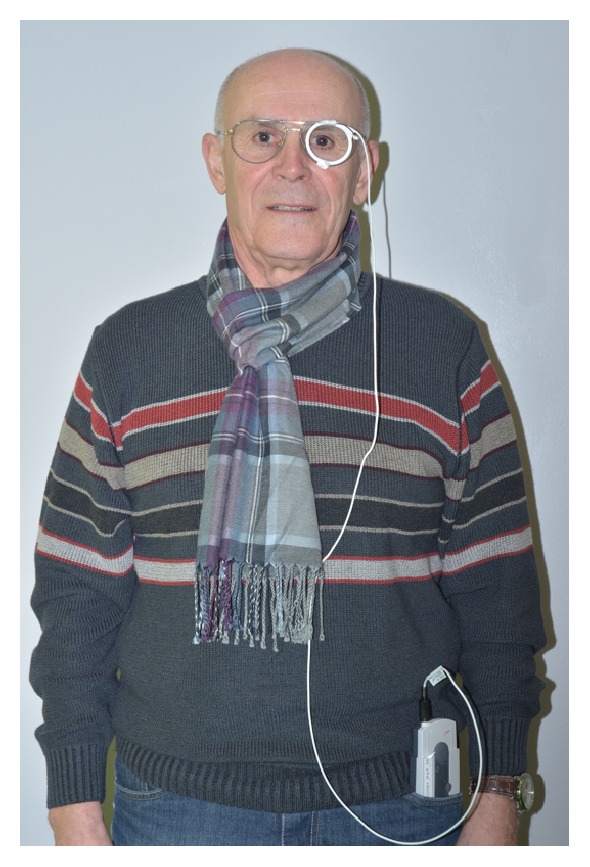
For improved wear comfort during daytime, the coil antenna was attached to an old eyeglass frame.

The patient did not experience any pain related to the measurement procedure. The only reported side effect was minor discomfort caused by the material of the sleep mask and the eyepatch. The patient attached the coil antenna to an old eyeglass frame to improve wearing comfort during daytime (Figure 3). During the measurements, there were no errors or other technical issues reported.

**Figure 4:**
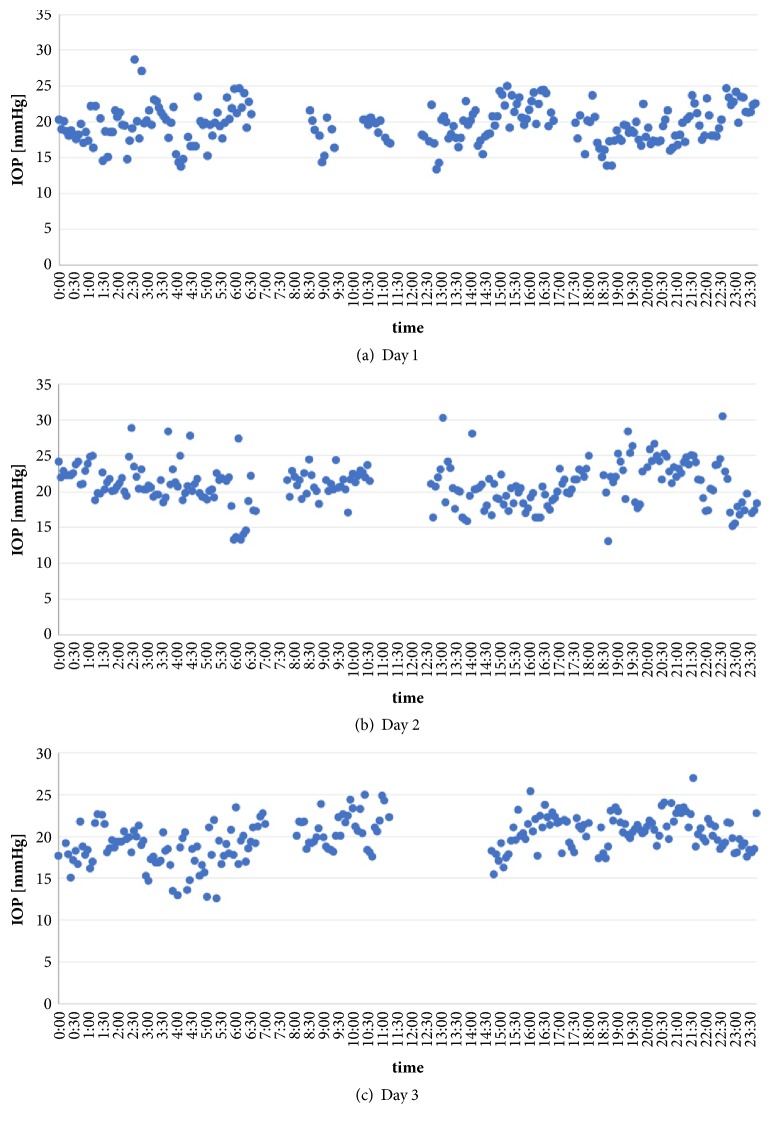
IOP (24 h profiles) of three consecutive days (a–c) with visualization of nyctohemeral IOP fluctuation.

The fluctuations in the readings for the three sequences of the 24 h IOP profiles are presented in Figures 4(a)–4(c).

The mean IOP, standard deviation, and range of readings were as follows: Sequence 1: 19.6 ± 2.7 mmHg, range 13.4–28.7 mmHg; Sequence 2: 21.0 ± 3.0 mmHg, range 13.1–30.5 mmHg; Sequence 3: 19.9 ± 2.4 mmHg, range 12.6–27 mmHg.

## 4. Discussion

Recently, Aptel et al. provided an overview of 24 h IOP monitoring devices and highlighted the importance of better understanding the nyctohemeral fluctuations in the IOP [13].

The major advantages of an intraocular sensor are the capability of a noncontact IOP measurement, being independent of corneal properties, ease of use, and the large number of measurements that can be performed during daytime and nighttime.

For the first time, repeated and automated 24 h noncontact IOP profiles are made possible using a prototype reading system after implantation of a novel telemetric IOP sensor (generation 1) in patients with glaucoma.

A similar measuring setup has been used in combination with a contact lens ocular telemetry sensor (Sensimed Triggerfish, Lausanne, Switzerland) that enables IOP monitoring over a period of 24 hours. In this approach, a bandage-fixed periorbital antenna is connected to a portable recorder. In previous studies, good tolerability and wear comfort were reported with this system. However, the wear comfort of this measuring system was evaluated only in combination with the contact lens and not independently [14–16].

Considering that the intraocular telemetric IOP sensor may be used for repeated, long-term measurements, special attention should be paid to the wear comfort of the measuring setup. Future studies should compare different systems, such as those mentioned in the present article, in order to evaluate tolerability and comfort using standardized questionnaires. The design of automated measuring systems and the materials used in these procedures should be optimized to improve wear comfort, quality of sleep, and patients' acceptance.

The present study was characterized by limitations that must be acknowledged. Firstly, this study included a single patient and, thus, had access to limited data. Secondly, as mentioned earlier, the accuracy of the IOP sensor was* per se* a limiting factor in the first-generation sensor system. A detailed analysis of the IOP data obtained in this present study, as well as a precise statement to the diurnal data, may not be adequate at this point as long as the accuracy of the sensor is not guaranteed.

Therefore, the investigators decided to concentrate their efforts on describing the feasibility of the automated measuring procedure. All data collected by the first-generation sensors have impacted the design of the next generation of sensors. Therefore, despite the involvement of a single patient in the present study, it may be useful to share these findings with the medical community.

Thirdly, there are no predefined questionnaires used for the evaluation of the measuring system. The interpretation of the results is influenced by the nonblinded investigator, which may lead to bias. In future studies, standardized questionnaires should be developed to improve the quality and power of the results. In addition, the use of a logbook to record all activities and the switch between measuring modalities (eyeglass frames, sleep masks, or other future options) during the study period may be useful.

The interpretation and analysis of future IOP sensor data will be a challenging task for glaucoma specialists. The availability of repeated 24 h IOP profiles with high temporal resolution may improve the follow-up of patients with glaucoma and the understanding of IOP fluctuations. An interesting future challenge for clinicians may be to identify the population among patients with glaucoma for whom this telemetric IOP sensor system should be recommended. The investigators of the present study suggest that patients with unclear visual field deterioration under seemingly well controlled IOP or those unable to visit an ophthalmologist regularly may benefit from this method.

As mentioned earlier in this article, concerns regarding the accuracy of the first-generation telemetric sensor and its ability to reliably measure the IOP have been expressed [10, 11]. The expected results of the ongoing clinical trials assessing the second-generation sensor may address these concerns prior to the introduction of this device into clinical practice.

## Data Availability

The data that support the findings of this study are available from the corresponding author upon reasonable request.
